# Microscopic Understanding of Interfacial Performance and Antifoaming Mechanism of REP Type Block Polyether Nonionic Surfactants

**DOI:** 10.3390/molecules29081816

**Published:** 2024-04-17

**Authors:** Yifei Zhao, Chunlong Xue, Deluo Ji, Weiqian Gong, Yue Liu, Ying Li

**Affiliations:** Key Lab. of Colloid and Interface Chemistry of State Education Ministry, School of Chemistry and Chemical Engineering, Shandong University, Jinan 250100, China

**Keywords:** propylene oxide, ethylene oxide, antifoam, interfacial molecular behaviors, molecular dynamics simulation, foam properties

## Abstract

In many practical applications involving surfactants, achieving defoaming without affecting interfacial activity is a challenge. In this study, the antifoaming performance of REP-type block polymer nonionic surfactant C12EOmPOn was determined, and molecular dynamics simulation method was employed to investigate the molecular behaviors of surfactants at a gas/water interface, the detailed arrangement information of the different structural segments of the surfactant molecules and the inter-/intra-interactions between all the structural motifs in the interfacial layer were analyzed systematically, by which the antifoaming mechanisms of the surfactants were revealed. The results show that the EO and PO groups of REP-type polyether molecules are located in the aqueous phase near the interface, and the hydrophobic tails distribute separately, lying almost flat on the gas/water interface. The interaction between the same groups of EOs and POs is significantly stronger than with water. REP block polyethers with high polymerization degrees of EO and PO are more inclined to overlap into dense layers, resulting in the formation of aggregates resembling “oil lenses” spreading on the gas/water interface, which exerts a stronger antifoaming effect. This study provides a smart approach to obtaining efficient antifoaming performance at room temperature without adding other antifoam ingredients.

## 1. Introduction

The application of foam is extensive in both daily life and industrial production, encompassing fields such as foam fire suppression, mineral flotation, enhanced oil recovery, detergents, and cosmetics [1,2,3,4]. But in many practical processes, foam generation is a double-edged sword; in some cases, the formation of foams can be harmful [5,6,7]. For instance, the presence of excessive foam can have detrimental effects on product quality, impede production efficiency, disrupt process operations, and potentially cause equipment damage, thereby posing safety hazards [5,8,9]. In the field of detergents, producing abundant foam used to be a major requirement for showing product performance in the hand-washing process. In the present day, the utilization of low-foam detergents is more favored, which can effectively shorten rinsing time, resulting in substantial water and electricity conservation while also reducing sewage discharge [10,11,12]. On many occasions like this, the achievement of effective antifoaming while maintaining the interfacial activity of the surfactant systems is of great importance, which is still a challenge.

The antifoaming phenomena are intrinsically related to defoaming. Basically, defoamers are utilized to destroy existing foam, while antifoamers are used to prevent foam generation [13]. Antifoamers can act as defoamers, while not all defoamers have the ability to prevent foaming. The two effects could be intersected in many situations, and effective applications of both effects need to be based on a clear understanding of the mechanisms [14], which are necessary to be further extended.

The currently used defoaming approaches can be broadly classified into two categories: physical and chemical methods. The physical methods mainly involve applying external forces to alter the physical states of the formed foam and cause its rupture, such as mechanical stirring, ultrasonic treatment, electrostatic action, and thermal treatment. [8,9,15,16]. The chemical methods typically employ chemical additives as defoamers, which has become the most commonly employed approach in modern industrial productions due to its advantages, such as rapid defoaming, prolonged duration, and cost-effectiveness [9]. Physical defoaming methods are hard to use in antifoaming. While most of the currently employed defoamers include mineral oil, fatty acids, fatty alcohols, surfactants, silicone compounds (silicone oil or polysiloxane), solid hydrophobic particles, etc. [6,17,18,19], might be antifoamfers, though the mechanisms of different types of defoamers are quite different. Reducing the solubility of surfactants is the most commonly used defoaming category. For example, the addition of oppositely charged electrolytes to anionic surfactant systems was demonstrated, in certain studies, to result in foam rupture [20,21]. Solid particles and oil droplets could exhibit a defoaming effect through a bridging-stretching mechanism, the spreading-fluid entrainment mechanism, and the spreading-wave generation mechanism [9,17,22,23]. 

In all the possible methods, the approaches only using surfactants without the need for additional antifoaming ingredients are more expected, while it is also very difficult to achieve and control. For example, the combination of cationic and anionic surfactants can lead to a defoaming effect by means of precipitation or phase separation resulting from the electrostatic interactions between the head groups [24,25], while the interfacial activity would be greatly reduced and even disappeared. Nonionic surfactants could exhibit defoaming properties at high temperatures above their cloud point; the condensed phase formed from the insoluble surfactants acts as oil droplets bridged onto the foam film, facilitating its defoaming action corresponding to temperature rising [26,27,28]. Maintaining the interfacial activity of the surfactant systems while inhibiting its foamability is a tricky problem and a big challenge, especially at room temperature.

In recent years, a new REP-type of block polyether nonionic surfactant has been developed. REP-type block polyethers are a class of polymers with epoxy structure, such as repeated units of ethylene oxide (EO) or propylene oxide (PO) groups, synthesized by polymerization reaction triggered by initiators, and their molecular structure is RO-(EO)_x_-(PO)_y_-H, where R stands for hydrophobic alkyl chain [29,30]. They have been employed as antifoamers in detergent formulations, showing many advantages, such as well-regulated molecular structure, the absence of precipitation, and cost-effectiveness [31]. Its capabilities in emulsification and solubilization processes also attracted some attention [32,33]. However, the related microscopic-level mechanisms are scarce, and their effective application is limited. Understanding the structure–performance relationship of such surfactants is meaningful for further optimization and the development of efficient antifoaming approaches.

In this paper, the equilibrium and dynamic surface activities and foam properties of pure REP-type nonionic surfactants and the compound systems with anionic surfactant sodium dodecyl sulfate (SDS) were experimentally determined, and molecular dynamic simulations were employed to elucidate the behavior of the surfactant molecules at the gas/water interface. The excellent foamability of SDS can introduce obvious differences in foam properties to the experimental results of the composite system. By analyzing the tilt angle of each component towards the z-axis, the hydrogen bonds between EO/PO chains and water, examining the radial distribution function, and investigating intermolecular interactions among different groups, a comprehensive understanding of the arrangement and aggregation behavior of REP-type polyether molecules at the gas/water interface was provided, by which the antifoaming mechanisms were analyzed. A common nonionic surfactant, AEO-9, with similar hydrophobic tails, was used as a reference. This work might provide a theoretical basis and guidance for the design and application of low foam systems with high surface activity.

## 2. Results and Discussion

### 2.1. Surface Activity and Foaming Properties of REP Type Block Polyether Nonionic Surfactants

**Figure 1:**
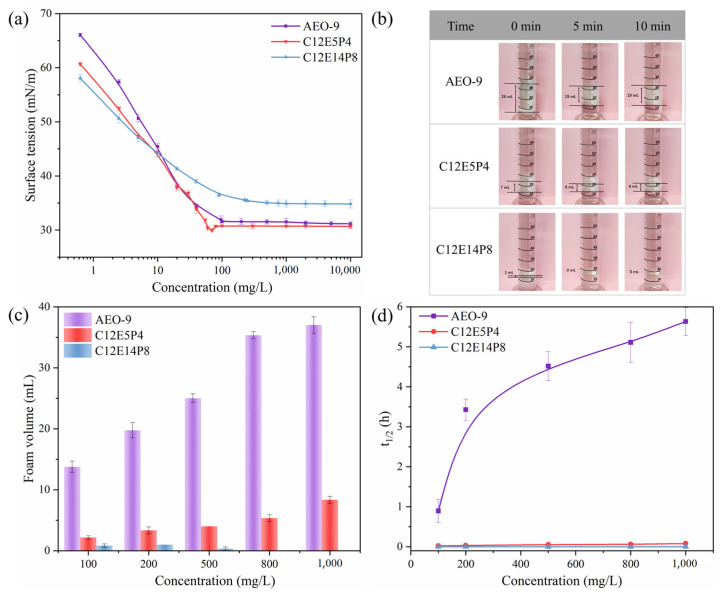
The surface activity and foam properties of AEO-9, C12E5P4, and C12E14P8 solutions. (**a**) The variation of equilibrium surface tension as a function of surfactant concentration at 298 K. (**b**) The photos of foam column taken at different times after foam generation at 298 K. The concentration of surfactant is 500 mg/L. (**c**) Foaming ability of different types of surfactants represented by the initial foam volume at 298 K. (**d**) Foam stability of different types of surfactants represented by foam half-life time t_1/2_ at 298 K.

The equilibrium surface tension curves of the three nonionic surfactants, AEO-9, C12E5P4, and C12E14P8, are shown in Figure 1a. The equilibrium surface activity parameters, such as the maximum surface excess (Γ_max_) and the minimum area per molecule (A_min_) of surfactant at the air–water interface, are calculated according to Equations (1) and (2), and these parameters are shown in Table 1.

(1) $$\Gamma_{\max}=-\frac{1}{2.303\mathrm{nRT}}{\left(\frac{d\gamma }{\mathrm{dlgc}}\right)}_{T}$$

(2) $$A_{\min}=\frac{1}{{N_{A}\Gamma }_{\max}}$$

According to the results in Figure 1a and Table 1, AEO-9 and C12E5P4 have similar γ_CMC_ and CMC. The structural difference between AEO-9 and C12E5P4 is not much, so it is reasonable their surface activity is similar.

Generally speaking, foam properties of surfactants are closely related to surface activity [25]. It can be seen from the results in Figure 1b–d that there is a significant difference in the foaming capacity and foam stability between AEO-9 and C12E5P4. The AEO-9 exhibits excellent foam stabilization capability, whereas the foam ability of C12E5P4 is low, and the generated foam bursts rapidly. It indicates that the introduction of four propylene oxide groups in the C12E5P4-replacing EO groups causes significant differences in foam properties, which is very interesting and provides perfect comparative systems to explore the influence of interfacial behavior of surfactants with different molecular structures on foam properties, which is further investigated in the later section.

Comparing the two REP-type nonionic surfactants, it can be observed that C12E14P8 exhibits a little larger CMC and higher γ_CMC_ compared to C12E5P4 and AEO-9, while the Γ_max_ is much larger and A_min_ is much smaller. Under the same surfactant concentration, the foaming ability and foam stability of C12E14P8 are far below AEO-9 and C12E5P4, showing strong antifoaming performance. The results demonstrate that an increase in the degree of polymerization positively impacts the antifoaming effect of REP-type nonionic surfactants.

According to the above results, it is evident that REP-type nonionic surfactants have the potential to be used as antifoamers. For the purpose of understanding the mechanisms clearly, the adsorption kinetics of the surfactants are experimentally determined, and the interfacial molecular behaviors are investigated by molecular dynamic simulations.

### 2.2. Dynamic Surface Activity of REP Type Block Polyether Nonionic Surfactants

**Figure 2:**
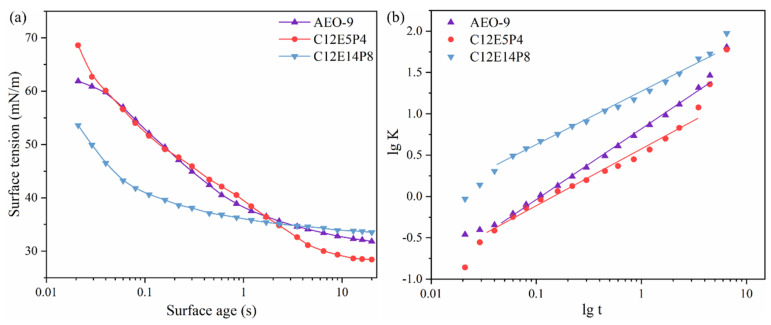
(**a**) Dynamic surface tension of samples under concentration of 500 mg/L at 298 K. (**b**) Dynamic surface tension parameters lgK as function of lgt for AEO-9, C12E5P4, and C12E14P8 at 298 K.

The time-dependent dynamic surface tension of AEO-9, C12E5P4, and C12E14P8 solutions under identical concentrations of 500 mg/L were measured, as shown in Figure 2a. The adsorption kinetics of the surfactants were analyzed using a model proposed by Rosen et al. [34].

The dynamic surface tension curves of the two nonionic surfactants, AEO-9 and C12E5P4, exhibit similar trends at this concentration, as depicted in Figure 2a, but are different from that of C12E14P8. The interpretation of the dynamic surface tension data are facilitated using the empirical equation proposed by Rosen et al. [35]:

(3) $$\frac{\gamma_{0}-\gamma_{t}}{\gamma_{t}-\gamma_{m}}={\left(\frac{t}{t^{*}}\right)}^{n}$$

The surface tension of the solvent is denoted as γ_0_, while the surface tension of the solution at time t is represented by γ_t_. The surface tension of the solution at equilibrium is expressed as γ_m_. The constant n quantifies the surfactant’s tendency to diffuse into the adsorbed layer. t* denotes the time required to reach half of the intermediate equilibrium value Π (Π = γ_0_ − γ_t_). Equation (4) is derived through a logarithmic transformation of Equation (3) [35]:

(4) $$\mathrm{lg}\frac{\gamma_{0}-\gamma_{t}}{\gamma_{t}-\gamma_{m}}=\mathrm{nlgt}-{\mathrm{nlgt}}^{*}$$

The adsorption energy of the adsorption process is mainly reflected by −nlgt*. The smaller the adsorption energy, the smaller t*, reflecting that the surfactant molecules are more easily adsorbed to the interface [36]. Equation (4) simplifies lg[(γ_0_ − γ_t_)/(γ_t_ − γ_m_)] to lgK, establishing a linear relationship with lgt as the independent variable and lgK as the dependent variable. Thus, the slope of the linear equation represents the constant n in Rosen’s formula, and the intercept represents −nlgt*.

R_1/2_ represents the rate at which the surface tension in the rapid descent zone drops at time t* [35]:

(5) $$R_{1/2}=\frac{\gamma_{0}-\gamma_{m}}{2t^{*}}$$

The faster the surface tension decreases, the greater the R_1/2_ value. The calculated values of n, t*, and R_1/2_ are shown in Table 2.

The t* of C12E14P8 is the smallest at the same concentration, indicating a lower adsorption barrier and stronger adsorption tendency. The time required for the surfactant molecules to diffuse from the bulk solution to reach the surface is the shortest and could reach an intermediate equilibrium state faster. A smaller value of n corresponds to a stronger diffusion trend. Comparing the parameters of these three nonionic surfactants reveals that, at this concentration, REP-type polyether exhibits a stronger diffusion trend than AEO-9, while C12E14P8 showed even stronger diffusion trends than C12E5P4, which suggests that introducing more propylene oxide groups facilitate the increase in the diffusion and interfacial adsorption of the nonionic surfactant molecules in aqueous solutions. Combined with the foam performance, it can be considered that the diffusion of REP-type polyether nonionic surfactant in the bulk phase and the adsorption on the gas/water interface has a certain relationship with the foam inhibition performance.

### 2.3. Molecular Behavior of REP Type Block Polyether Nonionic Surfactants at Gas/Water Interface

**Figure 3:**
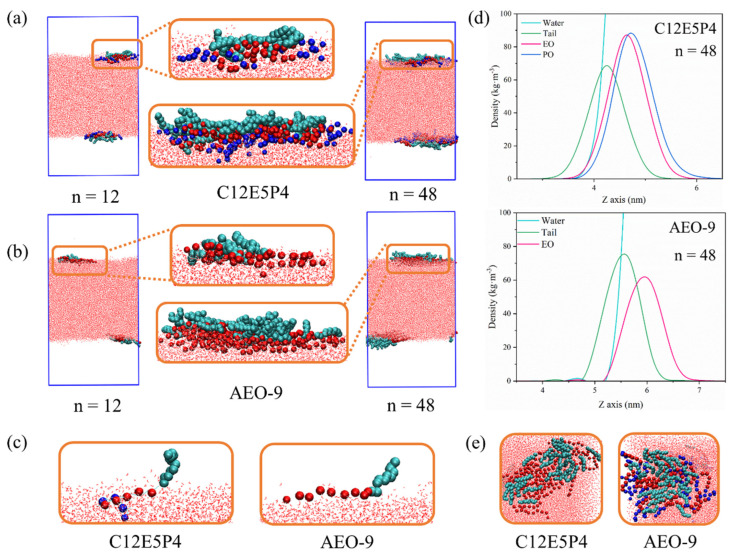
The side views of MD-simulated equilibrium configurations of (**a**) C12E5P4 and (**b**) AEO-9 with different molecular numbers at 298 K. (**c**) Conformation of one C12E5P4 molecule and one AEO-9 molecule at the gas/water interface extracted from (**a**,**b**). (**d**) Density of carbon chain and head groups of C12E5P4 and AEO-9. (**e**) The top views of MD simulated equilibrium configurations of C12E5P4 and AEO-9 (n = 48). (The green ball represents the C atom on the tail chain of hydrophobic carbon; the red ball represents the O atom in ethylene oxide groups; the blue balls represent the O atoms in propylene oxide groups).

A comparative analysis of molecular behaviors of REP nonionic surfactants at the gas/water interface in foam films was conducted using molecular dynamic simulations. The equilibrium configurations of foam films with different mass concentrations of C12E5P4 and AEO-9 are shown in Figure 3. In the snapshot containing a smaller number of molecules, the arrangement behavior of single characteristics could be observed, while in the snapshots containing a larger number of molecules, the effect of inter-molecular interaction on the molecular behaviors could be noticed.

Figure 3a–c show the side view of the MD simulation snapshots of C12E5P4 and AEO-9. The density distribution of the head group perpendicular to the gas/water interface plane along the z-axis was calculated to gain further insights into the structural properties of the head group, as shown in Figure 3d. It could be found that C12E5P4 and AEO-9 molecules both adsorb on the interface with their hydrophobic carbon tail chains extending into the gas phase, and EO chain segments are located near the interface in the aqueous phase, within a depth of 2 nm for both AEO-9 and C12E5P4. When the number of molecules increased, the EO groups of C12E5P4 and AEO-9 both tended to aggregate due to the strong interactions between EO groups. It was very interesting that the PO group was commonly considered hydrophobic, while the PO chains of C12E5P4 are located near the interface in the aqueous phase, too. Because C12E5P4 is terminated by hydroxyl (-OH), which is more hydrophilic than the EO group, the PO chain is pulled a little deeper into the water phase than the EO groups. Another detail that needs to be mentioned is that propylene oxide has a branched methyl group (-CH_3_), resulting in significant steric hindrance that impedes aggregation between PO groups, so the PO segments aggregate looser than EO groups, so C12E5P4 take up more space on the interface than AEO-9 when the number of molecules on the interface increases, which coincide with the results in Table 1.

**Figure 4:**
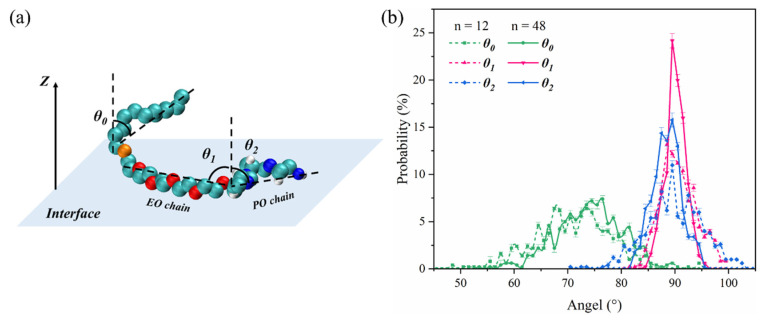
(**a**) Definition of the tilt angle of carbon chain, EO chains, and PO chains, θ_0_, θ_1_, and θ_2_ are the tilt angles of carbon chain, EO chains, and PO chains. (**b**) Distribution probability of different tilt angles in two concentration systems of C12E5P4 at 298 K.

The tilt angles (of which the definition was shown in Figure 4a) of the carbon chain, EO chain, and PO chain of C12E5P4 with z-axes were calculated to describe more clearly the conformation of REP-type nonionic surfactants on the interface, as shown in Figure 4b. θ_0_ is relatively chaotic and disordered along the direction of the gas/water interface, ranging from 50° to 90°; even when the concentration increased, θ_0_ did not change even a little, which is obviously different from the commonly excellent conformation of the hydrophobic tails of the regular surfactants. θ_1_ is kept at approximately 90°; the increase in the concentration of surfactant molecules leads to a gradual reorientation of θ_1_, becoming a little more perpendicular to the z-axis. θ_2_ undergoes similar changes to θ_1_. It could be concluded that the EO and PO segments eventually remain parallel to the gas/water interface, lying almost flat, though θ_2_ exhibits a little wider distribution range. The EO and PO chains exhibit the stretching state parallelly to the interface and occupy a large interfacial area, so the hydrophobic carbon tails could only lie flat without the opportunity to interact with each other, and that is why the stability of C12E5P4 foam decreased, as shown in Figure 1.

**Figure 5:**
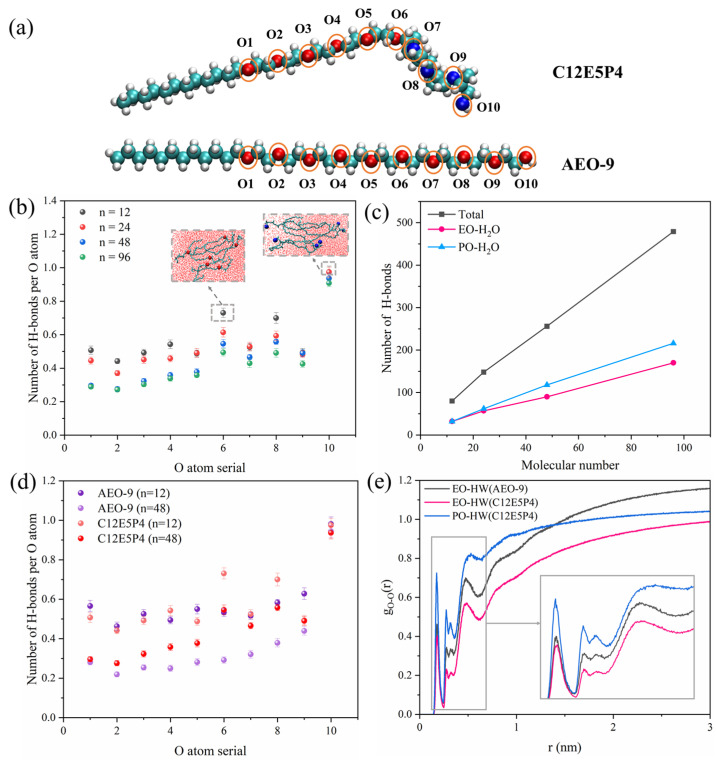
(**a**) Diagram of oxygen atomic number of C12E5P4 and AEO-9. (**b**) The average number of hydrogen bonds per oxygen atom in C12E5P4 system under different concentrations. (**c**) Hydrogen bonds change of C12E5P4 systems with different molecular numbers at 298 K. (**d**) The average number of hydrogen bonds per oxygen atom in C12E5P4/AEO-9 system under different concentrations at 298 K. (**e**) Radial distribution functions of ether groups and water at different positions at 298 K.

The quantification of hydrogen bonds formed between surfactants and water molecules was conducted along the PEO-PPO structure extending to the -OH, and the results are shown in Figure 5. It was found that two minor peaks are observed at the positions of O_6_ and O_8_, primarily due to the fact that O_6_ is located at the end of the EO chain, where there is folding in the PO chain and weak aggregation between EO molecules, allowing this oxygen atom to be more exposed to the aqueous environment. Similarly, a significant molecular gap exists at position O_8_ as well. The O_10_ in the -OH position forms the highest number of hydrogen bonds with water. As the number of molecules in the system increases, the average number of hydrogen bonds that each O atom forms with water decreases except O_10_, corresponding to the inter- or intra-molecular aggregation of EO and PO groups. When n = 12, EO forms an equal number of hydrogen bonds with water as PO, and the aggregation degree of EO and PO groups are both weak. When n > 12, the clustering of EO groups is clearer than PO because the interaction between EO is stronger, so PO groups form more hydrogen bonds with water than the EO.

**Figure 6:**
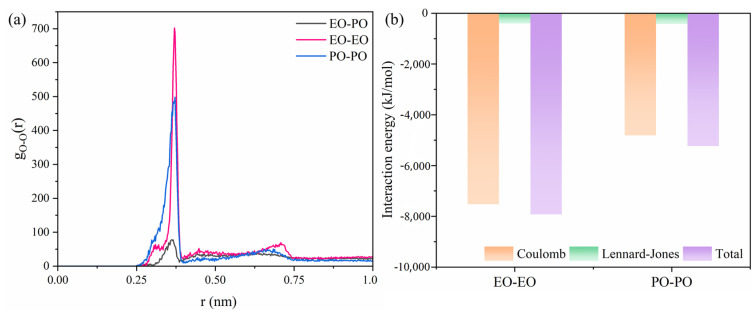
(**a**) Radial distribution functions of EO–PO groups, EO–EO groups, and PO–PO groups in C12E5P4 system (n = 48) at 298 K. (**b**) Coulomb interaction potential and Lennard-Jones potential between different groups in C12E5P4 system (n = 48) at 298 K.

As for the results of AEO-9, which are shown in Figure 5c, the number of hydrogen bonds formed by individual oxygen atoms in AEO-9 with water is fewer than that of C12E5P4. Figure 5d illustrates variations in radial distribution functions for ether groups and water molecules at different positions within the C12E5P4 system and the AEO-9 system. The first peak of these curves is observed at 0.18 nm. The radial distribution function value of the first peak for -O-(PO) is 0.72, while the second, third, and fourth peaks occur at 0.28 nm, 0.32 nm, and 0.51 nm, respectively. The peaks of EO occur in approximately the same positions as PO, but they are weaker overall. With the increase in the number of C12E5P4 molecules, the interaction between EO and PO with water is weakened, and the influence on EO groups is greater (Figure S4). The above results suggest that the affinity between EO groups drives the close aggregation of EO groups, while the interaction between PO groups is weaker, which agrees well with the results shown in Figure 6. A comparison of the RDF peaks in Figure 6a shows that EO-EO interactions exhibit the highest intensity with a peak value of approximately 700, while PO-PO interactions are comparatively weaker with a peak value of 500. In addition, EO-PO groups display the weakest affinity, characterized by a peak value of 75. The results prove that the interaction between EO groups is stronger, and the aggregation trend is stronger. The interaction energy between different groups is given in Figure 6b. It can be found that the interaction between the EO or PO groups is primarily dominated by the Coulomb force, which is much stronger than van der Waals force, and the Coulomb interaction energy between EO-EO groups is surely greater than that between PO-PO groups.

Based on the preceding discussion, it can be inferred that the interaction between PO-PO groups is weaker than EO-EO groups, and the interaction between EO-PO groups is the weakest. The C12E5P4 occupies a larger area than AEO-9, and the hydrophobic tails of C12E5P4 could not intersect with each other to form a compacted stable interface layer like AEO-9, so the foam stability is low. 

**Figure 7:**
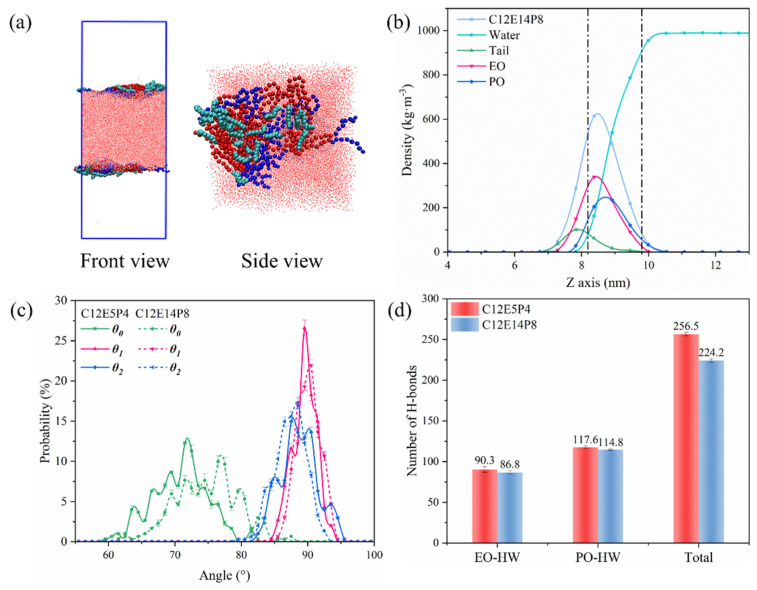
(**a**) The MD simulated equilibrium configuration of C12E14P8 (n = 48) at 298 K. (**b**) Density distribution of C12E14P8 under the concentration of 5 wt% at 298 K (Dotted line: interfacial thickness). (**c**) Probability distribution of tilt angle of different types of REP block polyether. (**d**) The number of hydrogen bonds formed between REP-type block polyether molecules and water.

The arrangement of C12E14P8 molecules at the interface differs somewhat from that of C12E5P4 and gave out important clues on improving the antifoaming performance of REP surfactant. Here, the simulation systems with the same mass concentration of 5 wt% were constructed, and since the molecular mass of C12E14P8 is about twice that of C12E5P4, the number of the former is half that of the latter. As shown in Figure 7a, the EO segments in C12E14P8 exhibit a more compact organization, while the PO chain tends to concentrate around the aggregate formed by the EO chains, too. The arrangement of C12E14P8 molecules is a little more loosely packed compared to that of C12E5P4, as depicted in Figure 7b, while the thickness of the C12E14P8 interface layer is undoubtedly thicker. The aggregation of C12E14P8 at the interface results in the formation of aggregates resembling “oil lenses”. According to the gyration radius shown in Table 3, it is observed that the size of C12E14P8 aggregates at the interface is larger than C12E5P4.

By analyzing the tilt angle, it can be observed from Figure 7c that the carbon chain of C12E14P8 is positioned closer to the gas/water interface compared to that of C12E5P4. The interaction between REP block polyether and water was examined by analyzing the number of hydrogen bonds, and the results are shown in Figure 7d. The total number of hydrogen bonds formed by C12E14P8 with water is smaller than that of C12E5P4, especially for the EO chain, of which the average hydrogen bond formed by each EO group with water is significantly reduced. This indicates that there is a substantial increase in EO aggregation as the degree of EO polymerization increases. Similar phenomena could be observed on the PO chain.

**Figure 8:**
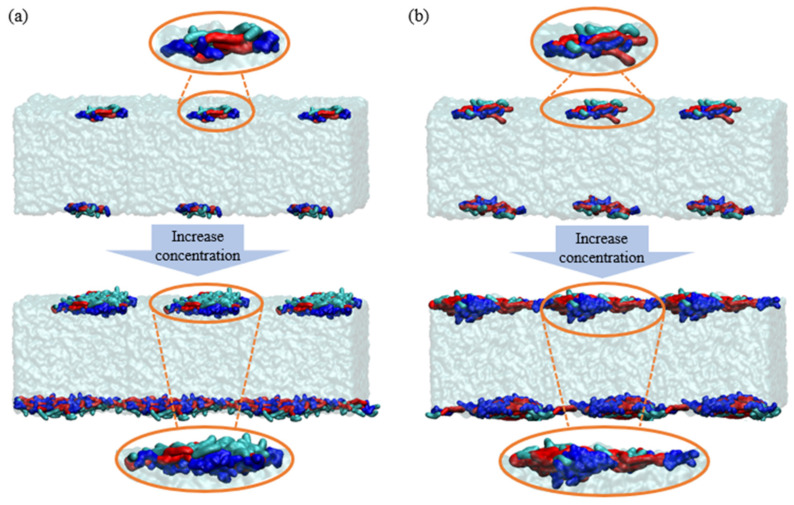
Distribution diagram of REP−type block polyether molecule aggregates on the foam liquid film: (**a**) C12E5P4 system; (**b**) C12E14P8 system. (Green segments represent carbon chain. Red segments represent EO chain. Blue segments represent PO chain).

The schematic diagram of C12E5P4 is depicted in Figure 8a. Compared with C12E5P4, with a lower degree of polymerization, and EO groups of C12E14P8, with a higher degree of polymerization, are more inclined to overlap into dense layers, and PO groups are arranged around the aggregates of EO to form larger “oil lens” at the interface (Figure 8b). The formation of an “oil lens” structure on the interface by the C12E14P8 molecules plays a role similar to oil droplets through a bridging-stretching mechanism [17], which is actually the key antifoaming mechanism of C12E14P8.

### 2.4. Interfacial Molecular Behavior and Foam Properties of Nonionic/Anionic Surfactant Binary Mixed System 

**Figure 9:**
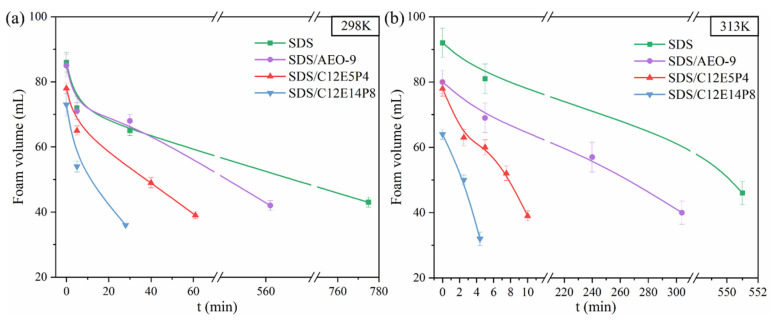
The variation of foam volume generated by pure SDS system and SDS compound system (mass concentration ratio is 4:1) with time (**a**) at 298 K and (**b**) at 313 K.

To further comprehend the antifoaming efficacy of REP surfactants in conjunction with other surfactants, foam determination experiments on pure SDS systems and SDS/nonionic surfactant compound systems were conducted. In the foam performance test, the mass concentration of pure SDS solution was 1000 mg/L, while the concentration of SDS in the binary composite system was 1000 mg/L, and the concentration of nonionic surfactant was 250 mg/L (the mass concentration ratio was 4:1). As depicted in Figure 9a, the foam formed from pure SDS system remains stable for a long time at room temperature, with a half-life time of up to 13 h. The SDS/AEO-9 system maintained similarly high foam stability. At the same time, the half-life time of the foam formed from the C12E5P4/SDS system is shortened to 1 h. C12E14P8 exhibited the best antifoaming effect in the compound systems, and the half-life time was shortened almost thirty times, reaching 28 min. At the common working temperature of a washing machine, 40 °C, the addition of C12E14P8 reduces the half-life of the compound SDS system by 110 times compared with pure SDS, as depicted in Figure 9b. This observation suggests that the incorporation of C12E14P8 significantly diminishes both the foaming ability and foam stability of the composite system, performing a nice antifoaming performance.

**Figure 10:**
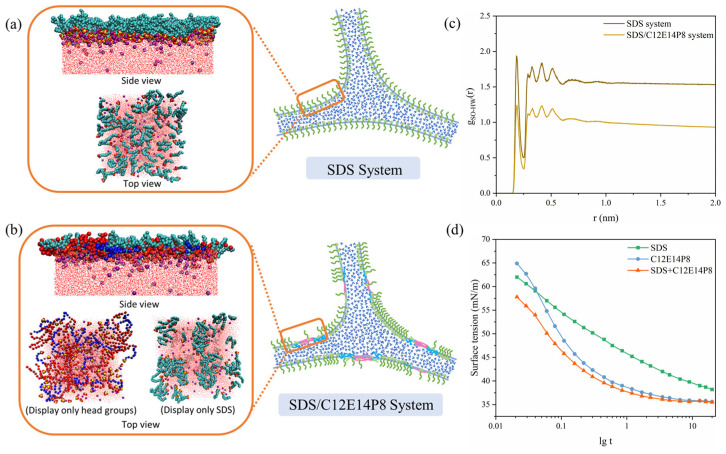
Top view of the molecular simulation snapshot and schematic diagram of (**a**) pure SDS foam liquid film; (**b**) C12E14P8 and SDS compound system. (**c**) Radial distribution functions of sulfuric acid groups and water in different systems at 298 K. (**d**) Dynamic surface tension curves of SDS system, C12E14P8 system, and SDS/C12E14P8 compound system at 298 K.

A molecular simulation was used to explore the antifoaming mechanism of C12E14P8 in the compound foam system at room temperature. In the pure SDS system, the SDS molecules exhibit a preference for upright orientation at the gas/water interface; with the sulfuric acid head groups penetrating into the aqueous phase, an ordered dense interface layer could be formed, even if there is electrostatic repulsion between the head groups (Figure 10a). In the compound system of C12E14P8 and SDS, C12E14P8 diffuses faster, occupying most of the interfacial area, forming dense aggregations like oil drops at the interface (Figure 10b), reducing the stability of the foam films. The RDF diagram (Figure 10c) also indicates that the existence of C12E14P8 weakens the interaction between sulfuric acid groups in SDS and water, simultaneously reducing the number of hydrogen bonds formed between sulfuric acid groups and water (Table S3). The results obtained from molecular simulation agree well with the foam determination experiments results. As it was shown by the dynamic surface tension curve of the compound surfactant in Figure 10d, the C12E14P8 does adsorb to the interface more rapidly than SDS. It is valuable to note that the interfacial activity of the C12E14P8/SDS compound system is higher than the SDS system and could be qualified as a low-foam system with high activity.

## 3. Material and Methods

### 3.1. Materials

**Figure 11:**
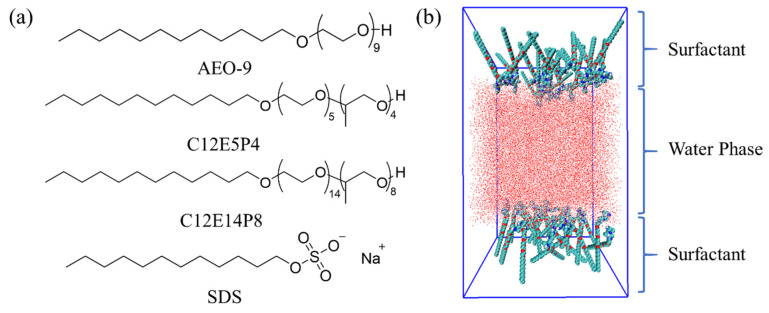
(**a**) Molecular structure of nonionic surfactant AEO-9, REP-type block polyether (C12E5P4 and C12E14P8) and anionic surfactant SDS. (**b**) Diagram of the initial state of the simulation system.

Nonionic surfactant dodecyl polyoxyethylene ether (AEO-9) (content ≥ 99.5%, M_w_~590) was purchased from BASF. The anionic surfactant sodium dodecyl sulfate (SDS) (AR, M_w_~288.38) was purchased from Macklin. Nonionic surfactant dodecyl polyoxyethylene polyoxyel ether C_12_H_25_O(EO)_m_(PO)_n_H (C12E5P4, m = 5, n = 4; C12E14P8, m = 14, n = 8, content ≥ 99.7%) were provided by Lianhong Company. The chemical structures of the surfactants are shown in Figure 11a.

### 3.2. Static Surface Tension

Sample solutions of different mass concentrations were prepared with ultra-pure water as solvent, and the surface tension was measured by Wilhelmy plate method using Krüss K100 surface tensiometer. The experimental temperature was controlled to be 25 ± 1 °C. Three groups were selected to record the data to reduce the error.

### 3.3. Foam Performance

Foaming capacity: The foaming capacity was tested by Bartsch shaking method [37]. The initial foam volume is used to indicate the foaming capacity. We added 15 mL of the prepared solution into a mixing cylinder with a stopper and quickly recorded the initial foaming height after hand-shaking for 30 s. We repeated the operation three times.

Foam stability: Static foam stability is expressed by half-life. The foam half-life refers to the time when the foam volume decays to half of the initial volume, recorded as t_1/2_. The above experimental temperature was controlled at 25 ± 1 °C.

### 3.4. Dynamic Surface Tension

Three different surfactant solutions (concentration 500 mg/L) were prepared with ultra-pure water as solvent. The dynamic surface tension was measured by SITA dynamic surface tensiometer (the maximum bubble pressure method) [38]. The temperature of the solution was controlled at 25 ± 1 °C.

### 3.5. Molecular Dynamic Simulation

The initial topologies of surfactants C12EmPn (Figure 11a) and SDS were optimized at B3LYP/6−31G(d) levels using the Gaussian16 software package [39]. The initial “sandwich” model of the surfactant/water system was established using the molecular packing program PACKMOL 17.125 [40]. As shown in Figure 11b, the initial configuration consists of 33,400 water molecules placed in a box with an intermediate size of 10.0 nm × 10.0 nm × 10.0 nm with different numbers of surfactant molecules on both sides of the water. In the anionic/nonionic surfactant complex system, in order to make the system electrically neutral, an equal amount of anti-ion (sodium ion) is randomly introduced into the water phase. All the systems and corresponding labels are listed in Tables S1 and S2.

All molecular dynamic calculations were performed in GROMACS 2019.6 [41]. The interatomic interaction is calculated based on the parameters and potential functions of the GROMOS 54A7 force field [42]. In this position, surfactant molecules are described by association atoms. In the simulation, the water molecules were described using the extended simple charge (SPC/E) model [43]. Periodic boundary conditions are applied to the x, y, and z axes of the box. The energy minimization step size is set to 10 fs, and the number of steps is set to 5000. Then, NVT simulation with step size of 2 fs was run for 10 ns for confirmation and analysis [44,45]. Berendsen thermostat was used to control the temperature, and the temperature was set at 298.15 K [46]. The key length is all limited by LINCS algorithm, and the truncation radius of the neighbor list is 1.2 nm, which is updated every 10 steps [47]. The Particle Mesh Ewald (PME) method was used to calculate the long-range electrostatic interaction with mesh spacing of 0.135 nm and truncation radius of 1.2 nm [34]. The analysis tools provided by GROMACS 2019.6 were used to analyze the simulation results, including the density distribution along the z-axis, radial distribution function, interface generation energy, and water molecular diffusion coefficient. Visualization and snapshot analysis of all molecular configurations using Visual Molecular Dynamics software (VMD) 1.9.3 [48]. According to Figures S1–S3, the simulation system reaches equilibrium at about 6 ns, and the trajectory of the last 4 ns is selected for data analysis.

## 4. Conclusions

In this paper, the surface activity, foam property, and molecular behavior of two REP-type block polyether nonionic surfactants, C12E5P4 and C12E14P8, at the gas/water interface was studied by combining experiment and molecular dynamics simulation methods for the purpose of better understanding the antifoaming mechanism. The experimental results show that REP-type polyether molecules have a high interfacial adsorption tendency, while the foaming property and foam stability are much lower than that of AEO-9. Among them, C12E14P8 has the most significant antifoaming performance and still shows an excellent antifoaming effect after mixing with a typical foam stabilizer SDS. The simulation results showed that REP-type poly-molecules adsorbed almost flat on the gas/liquid interface, the EO and PO groups were all located in the water phase near the interface, and the hydrophobic tails distributed separately, lying almost flat on the gas/water interface. A radial distribution function and energy analysis showed that the interaction between the same kind of groups as EOs and POs is significantly stronger than that between the EO and PO groups and water. When the degree of polymerization of EO and PO increases, the EOs and POs aggregate separately to form structures similar to an “oil lens”, so the foam liquid film has low stability and poor foaming ability. In the composite system with SDS, the interfacial adsorption rate of polyether molecules is faster than that of SDS, occupying the interfacial sites, which reduces the adsorption capacity and foam stability of SDS in the composite system, so it shows remarkable antifoaming performance. This study provides valuable insights for designing low-foam systems with high interface activity with bright application potentials in many related industries, such as washing.

## Figures and Tables

**Table 1:** Values of critical micelle concentration (CMC), equilibrium surface tension at CMC (γ_CMC_), surface excess (Γ_max_), and minimum area per molecule (A_min_) for surfactant AEO-9, C12E5P4, and C12E14P8 at 298 K.

Surfactant	CMC (mg/L)	γ_CMC_ (mN/m)	Γ_max_ (μmol/m^2^)	A_min_ (nm^2^)
AEO-9	94.64	31.54	2.660	0.63
C12E5P4	63.46	30.77	2.605	0.64
C12E14P8	246.69	34.85	1.190	1.40

**Table 2:** Dynamic surface tension parameters of AEO-9, C12E5P4, and C12E14P8 under concentration of 500 mg/L at 298 K.

Surfactant	n	t* (s)	t_i_ (s)	t_m_ (s)	R_1/2_ (mN m^−1^ s^−1^)
AEO-9	0.824	0.106	6.47 × 10^−3^	1.72	185.6
C12E5P4	0.668	0.152	4.83 × 10^−3^	4.75	140.8
C12E14P8	0.652	0.012	3.40 × 10^−4^	0.40	1643.7

**Table 3:** Interfacial thickness and gyration radius (R_g_) of different systems with same concentration (5 wt%) at 298 K.

Surfactant	Interface Thickness (nm)	R_g_ (nm)
C12E5P4	0.76	6.21
C12E14P8	1.61	7.08

## Data Availability

Data are contained within the article and Supplementary Materials.

## Supplementary Materials

The following supporting information can be downloaded at: https://www.mdpi.com/article/10.3390/molecules29081816/s1, Figure S1: RMSD of SDS/C12E14P8 binary compounding system; Figure S2: (a) Density distributions of AEO-9 and C12E5P4 (n = 48) at 6−7 ns and 9−10 ns, respectively; (b) The change of inclination Angle of C12E5P4 with simulation time at two concentrations of n = 12 and n = 48; (c) Density distributions of C12E14P8 at 6−7 ns and 9−10 ns, respectively; Figure S3: The total number of hydrogen bonds in C12E5P4 system with different molecular number changes with simulation time; Figure S4: Radial distribution functions of ether groups and water in (a) EO groups and (b) PO groups in C12E5P4 systems under different concentrations; Table S1: Summaries of simulated pure polyether systems; Table S2: Summaries of simulated SDS system and SDS/C12E14P8 system; Table S3: The number of hydrogen bonds formed between sulfuric acid groups and water in different systems at 298 K.
