# Supplementary material for: Microscopic Understanding of Interfacial Performance and Antifoaming Mechanism of REP Type Block Polyether Nonionic Surfactants
Source: Molecules. 2024 Apr 17;29(8):1816. doi: 10.3390/molecules29081816 (PMC11054078; doi:10.3390/molecules29081816)
Supplement: Supplementary file 1 [file molecules-29-01816-s001.zip › molecules-2924209-supplementary.pdf]

Supplementary Information

Microscopic Understanding of Interfacial Performance and Antifoaming Mechanism of REP Type Block Polyether Nonionic Surfactants

Yifei Zhao, Chunlong Xue, Deluo Ji, Weiqian Gong, Yue Liu and Ying Li *

Key Lab. of Colloid and Interface Chemistry of State Education Ministry, School of Chemistry and Chemical Engineering, Shandong University, Jinan 250100, China
* Correspondence: yingli@sdu.edu.cn

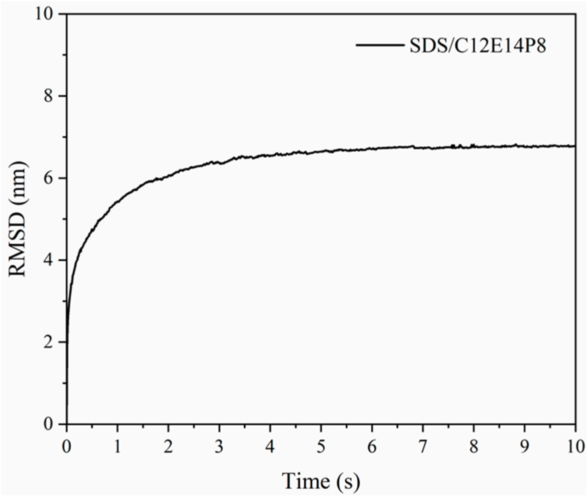

Figure S1. RMSD of SDS/C12E14P8 binary compounding system.

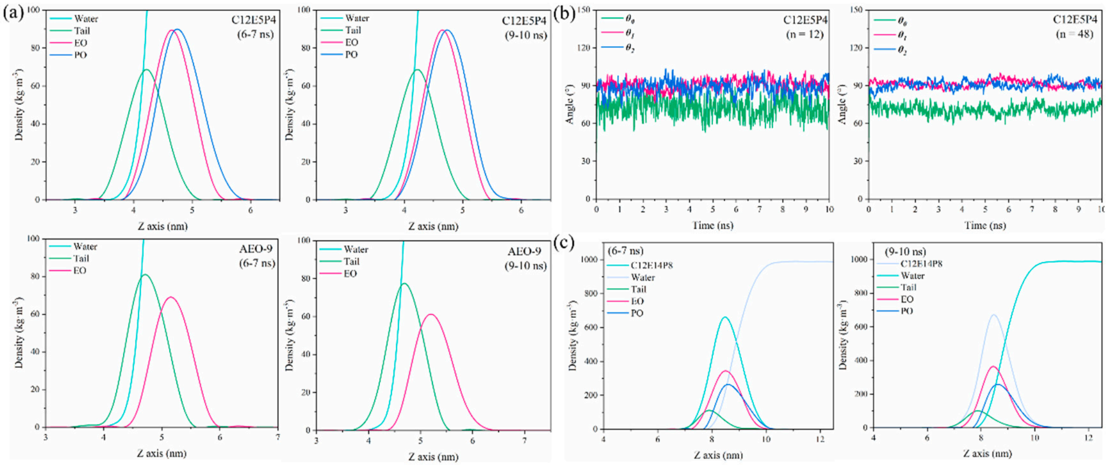

Figure S2. (a) Density distributions of AEO-9 and C12E5P4 (n=48) at 6–7 ns and 9–10 ns, respectively; (b) The change of inclination Angle of C12E5P4 with simulation time at two concentrations of n=12 and n=48; (c) Density distributions of C12E14P8 at 6–7 ns and 9–10 ns, respectively.

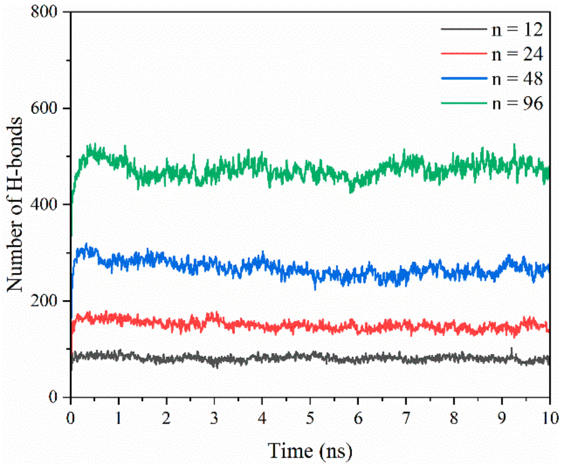

Figure S3. The total number of hydrogen bonds in C12E5P4 system with different molecular number changes with simulation time.

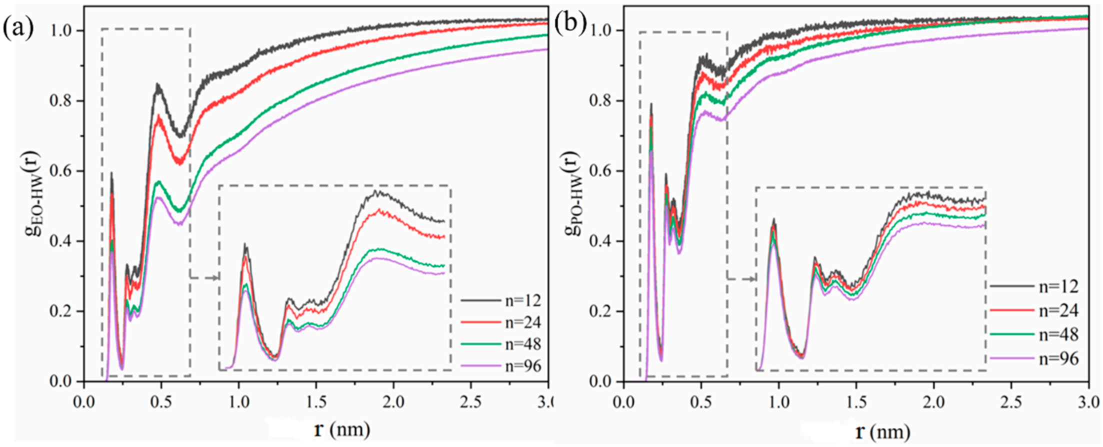

Figure S4. Radial distribution functions of ether groups and water in (a) EO groups and (b) PO groups in C12E5P4 systems under different concentrations.

Table S1. Summaries of simulated pure polyether systems.

System	Number of water	Number of polyether	Size of simulated box
C12E5P4 (n=12)	33400	12	$10 \times 10 \times 18$
C12E5P4 (n=24)	33400	24	$10 \times 10 \times 18$
C12E5P4 (n=48)	33400	48	$10 \times 10 \times 18$
C12E5P4 (n=96)	33400	96	$10 \times 10 \times 18$
AEO-9 (n=12)	33400	12	$10 \times 10 \times 18$
AEO-9 (n=48)	33400	48	$10 \times 10 \times 18$
C12E14P8 (n=48)	33400	48	$10 \times 10 \times 27$

Table S2. Summaries of simulated SDS system and SDS/C12E14P8 system.

System	Number of SDS	Number of C12E14P8	Number of water	Number of Na^+	Size of simulated box
SDS	192	-	33208	192	$10 \times 10 \times 15$
SDS/C12E14P8	192	48	33208	192	$10 \times 10 \times 27$

Table S3. The number of hydrogen bonds formed between sulfuric acid groups and water in different systems at 298 K.

System	Number of H-bonds (SO-HW)
SDS	1346.3
SDS/C12E14P8	1126.3
